# Genomic investigation unveils colistin resistance mechanism in carbapenem-resistant *Acinetobacter baumannii* clinical isolates

**DOI:** 10.1128/spectrum.02511-23

**Published:** 2024-01-12

**Authors:** Saranya Vijayakumar, Rayapadi G. Swetha, Yamuna Devi Bakthavatchalam, Karthick Vasudevan, Baby Abirami Shankar, Agilandeeswari Kirubananthan, Kamini Walia, Sudha Ramaiah, Indranil Biswas, Balaji Veeraraghavan, Anand Anbarasu

**Affiliations:** 1 Department of Clinical Microbiology, Christian Medical College, Vellore, Tamil Nadu, India; 2 Medical and Biological Computing Laboratory, School of Biosciences and Technology, Vellore Institute of Technology (VIT), Vellore, Tamil Nadu, India; 3 Department of Biotechnology, School of Applied Sciences, REVA University, Bangalore, India; 4 Division of Epidemiology and Communicable Diseases, Indian Council for Medical Research, New Delhi, India; 5 Department of Microbiology, Molecular Genetics and Immunology, University of Kansas Medical Center, Kansas, USA; Indian Institute of Technology Hyderabad, Hyderabad, Telangana, India

**Keywords:** *Acinetobacter baumannii*, colistin, resistance, SNP, *lpx*, *pmr*

## Abstract

Colistin resistance in *Acinetobacter baumannii* is mediated by multiple mechanisms. Recently, mutations within *pmrABC* two-component system and overexpression of *eptA* gene due to upstream insertion of IS*Aba1* have been shown to play a major role. Thus, the aim of our study is to characterize colistin resistance mechanisms among the clinical isolates of *A. baumannii* in India. A total of 207 clinical isolates of *A. baumannii* collected from 2016 to 2019 were included in this study. Mutations within lipid A biosynthesis and *pmrABC* genes were characterized by whole-genome shotgun sequencing. Twenty-eight complete genomes were further characterized by hybrid assembly approach to study insertional inactivation of *lpx* genes and the association of IS*Aba1-eptA*. Several single point mutations (SNPs), like M12I in *pmrA*, A138T and A444V in *pmrB*, and E117K in *lpxD,* were identified. We are the first to report two novel SNPs (T7I and V383I) in the *pmrC* gene. Among the five colistin-resistant *A. baumannii* isolates where complete genome was available, the analysis showed that three of the five isolates had IS*Aba1* insertion upstream of *eptA*. No *mcr* genes were identified among the isolates. We mapped the SNPs on the respective protein structures to understand the effect on the protein activity. We found that majority of the SNPs had little effect on the putative protein function; however, some SNPs might destabilize the local structure. Our study highlights the diversity of colistin resistance mechanisms occurring in *A. baumannii,* and IS*Aba1*-driven *eptA* overexpression is responsible for colistin resistance among the Indian isolates.

**IMPORTANCE:**

*Acinetobacter baumannii* is a Gram-negative, emerging and opportunistic bacterial pathogen that is often associated with a wide range of nosocomial infections. The treatment of these infections is hindered by increase in the occurrence of *A. baumannii* strains that are resistant to most of the existing antibiotics. The current drug of choice to treat the infection caused by *A. baumannii* is colistin, but unfortunately, the bacteria started to show resistance to the last-resort antibiotic. The loss of lipopolysaccharides and mutations in lipid A biosynthesis genes are the main reasons for the colistin resistance. The present study characterized 207 *A. baumannii* clinical isolates and constructed complete genomes of 28 isolates to recognize the mechanisms of colistin resistance. We showed the mutations in the colistin-resistant variants within genes essential for lipid A biosynthesis and that cause these isolates to lose the ability to produce lipopolysaccharides.

## INTRODUCTION

*Acinetobacter baumannii* is a major nosocomial pathogen that is responsible for a wide range of infections and has been reported as a global public health problem (1, 2). *A. baumannii* has developed resistance toward many different commonly used antibiotics, which results in the paucity of treatment options against *Acinetobacter* infections (3). Carbapenems are considered to be the last-line antibiotics for treating *A. baumannii* infections, and more than 80% resistance to carbapenem was reported (4). More than 60% mortality rates have been reported for the most common carbapenem-resistant *A. baumannii* (CRAB) infections, including bloodstream infections and hospital-acquired pneumonia (5).

Recently, the World Health Organization has categorized CRAB as a “Priority one” pathogen in the global list of antibiotic-resistant bacteria for the development of new antibiotics (3). The most common antibiotics considered for the treatment of CRAB include colistin-based, tigecycline-based, and sulbactam-based combinations (6). Unfortunately, increased usage of colistin for treating CARB infections resulted in the surge of colistin resistance (7).

In *A. baumannii*, colistin resistance is mediated by multiple mechanisms (8) such as complete loss of lipopolysaccharide (LPS) production by inactivation of the biosynthetic pathway and point mutations in lipid A biosynthesis genes, such as *lpxA*, *lpxC,* and *lpxD* (9, 10). In addition, the point mutations in the genes encoding the pmrCAB two-component system (TCS) also result in decreased membrane permeability and lead to increased colistin resistance in *A. baumannii* (11). The resistance mechanism of *pmr*CAB is due to the addition of phosphoethanolamine (pEtN) to LPS, which reduces the negative charge in the cell membrane that further prevents interaction with colistin. Moreover, insertion of IS*Aba1* at the upstream of the *eptA* gene leads to overexpression that attributes for increased colistin resistance (3). Importantly, plasmid-mediated colistin resistance due to the *mcr* gene that encodes pEtN transferase, which is involved in the efflux of colistin, has been reported in *A. baumannii* (12).

In this study, we characterized 207 *A*. *baumannii* clinical isolates to understand the molecular mechanisms of colistin resistance. We also generated complete genomes of 28 isolates to investigate the inactivation of *lpxA* or *lpxC* genes by IS*Aba11* insertion and to decipher the presence of IS*Aba1* element upstream of the *eptA* gene. Our results indicate that insertion of IS*Aba1* element to the upstream of *eptA* gene could be one of the major mechanisms for the occurrence of increased colistin resistance among Indian isolates.

## MATERIALS AND METHODS

### Bacterial isolates

A total of 1,214 consecutive non-duplicated clinical isolates of *A. baumannii* were collected during 2016 to 2019 as a part of routine diagnosis at Christian Medical College, Vellore, India. Among 1,214, 314 isolates were from blood and 900 isolates from endotracheal aspirate (ETA). All the isolates were identified at the species level as *Acinetobacter baumannii calcoaceticus* complex (*Acb* complex) using conventional biochemical methods (13). Matrix-assisted laser desorption ionization–time-of-flight mass spectrometry was used to confirm at the species level as *A. baumannii*. Further confirmation of *Acb* complex as *A. baumannii* was performed targeting chromosomally encoded *bla*_OXA-51_-like gene by PCR (14). Only carbapenem-resistant *A. baumannii* isolates were included in this study.

### Antimicrobial susceptibility testing

Antimicrobial susceptibility testing (AST) was performed for all the isolates against different classes of antibiotics by the Kirby Bauer disc diffusion method and interpreted according to the Clinical Laboratory Standards Institute guidelines (15). The antibiotics evaluated for the study are ceftazidime (30 µg), cefepime (30 µg), piperacillin-tazobactam (100/10 µg), cefoperazone-sulbactam (75/30 µg), imipenem (10 µg), meropenem (10 µg), levofloxacin (5 µg), amikacin (30 µg), netilmicin (30 µg), tobramycin (10 µg), aztreonam (30 µg), tetracycline (30 µg), minocycline (30 µg), and tigecycline (15 µg).

### Minimum inhibitory concentration by broth micro dilution

Minimum inhibitory concentration (MIC) value for colistin was determined for all the isolates using broth micro dilution (BMD) and interpreted accordingly (16). *Escherichia coli* ATCC 25922 and *Pseudomonas aeruginosa* ATCC 27853 were used as quality control (QC) strains. An *E. coli mcr-1*-positive isolate was used as an internal control. Two in-house *Klebsiella pneumoniae* QC strains BA38416 and BA25425 with colistin MIC values of 0.5 and 16 µg/mL, respectively, were also included in every batch of testing.

### Whole-genome sequencing, assembly, and annotation

Among the 1,214 strains, a subset of 207 *A*. *baumannii* isolates (blood = 103, ETA = 103, and pus = 1) were selected based on the source for further characterization by whole-genome sequencing (WGS). The genomic DNA was extracted using QIAamp DNA Mini Kit (QIAGEN, Germany) according to the manufacturer’s instructions, and WGS was performed. In brief, short-read sequencing was performed for all 207 isolates using IonTorrent Personal Genome Machine (Life Technologies, USA) with 400-bp read chemistry or by Illumina MiSeq as per the manufacturer’s instructions. Furthermore, a subset of 28 isolates (blood, *n* = 20; ETA, *n* = 7; and pus, *n* = 1) were selected based on the International Clones (ICs )and fewer novel sequence types (STs) for long-read sequencing. The long-read sequencing was performed using the SQK-LSK108 Kit R9 version (Oxford Nanopore Technologies, UK) using the 1D sequencing method according to the manufacturer’s protocol. To obtain complete genome, a hybrid assembly was performed on 28 genomes as described previously (17).

All the genomes were assembled and annotated using the National Center for Biotechnology Information (NCBI) Prokaryotic Genome Annotation Pipeline (18). Furthermore, a downstream analysis was performed using tools from the Center for Genomic Epidemiology server (http://www.genomicepidemiology.org/). Antimicrobial resistance genes were identified using the ResFinder 3.0 database (https://cge.food.dtu.dk/services/ResFinder/) (19). ST of the isolates was assigned by the MLST 2.0 tool using the Oxford scheme (*gltA*, *gyrB*, *gdhB*, *recA*, *cpn60*, *gpi*, and *rpoD* genes) (https://cge.food.dtu.dk/services/MLST/) (20).

### Mutation analysis of *lpxACD* and *pmrCAB* genes

The *lpxA*, *lpxC*, *lpxD*, *pmrA*, *pmrB*, and *pmrC* sequences were extracted from whole genomes of *A. baumannii* isolates, and an *in silico* mutation analysis was conducted in all the genomes using BLAST analysis (https://blast.ncbi.nlm.nih.gov) and compared with the reference strain of *A. baumannii* ATCC 17978 (GenBank accession number CP000521). Other *A. baumannii* reference strains, such as AYE (GenBank accession number NC010410), ACICU (GenBank accession number NC010611), and ATCC 19606 (GenBank accession number CP046654), were included in the analysis to identify the genetic polymorphisms. Detection of *mcr* genes was performed using either ResFinder or *in silico* BLAST analysis against the reference sequences of *mcr* genes reported so far (21). In addition to the mutation analysis, all the 28 complete genome sequences were characterized manually for other colistin resistance mechanisms including inactivation of *lpxA* and *lpxC* genes due to insertion of IS*Aba11* element and overexpression of *pmrC* homolog, *eptA,* due to upstream insertion of IS*Aba1* element.

### *In silico* analysis of protein structures by modeling

The protein sequences of LpxA, LpxC, LpxD, PmrA, PmrB, and PmrC were subjected to protein BLAST search against Protein Data Bank (PDB) (22) to verify the availability of their three-dimensional (3D) structures. The protein BLAST returned 100% identity with LpxA and PmrA sequences but not with the other proteins. Therefore, PDB has the structural information of LpxA (PDB ID: 4E6U) and PmrA (PDB ID: 7MUS); the 3D structures were downloaded in PDB format. The 3D structures of LpxC (AF-A3M9 × 5-F1) and LpxD (AF-A3M650-F1) were obtained from the AlphaFold protein structure database and retrieved. No structural information regarding PmrB and PmrC is currently available. To perform *in silico* protein modeling, iterative threading assembly refinement (I-TASSER) was used to predict the 3D structures of PmrB and PmrC (23). I-TASSER generated five different models, and the confidence of each model is quantitatively measured by C-score. which typically ranges from −5 to 2. C-score is a confidence score that was calculated based on the significance of threading template alignments and the convergence parameters of the structure assembly simulations. A higher C-score value signifies a model with a high confidence and vice versa. The mutant-type (MT) protein structures were obtained by replacing the specific point mutations in their respective wild-type (WT) protein structures using the Swiss PDB viewer package (24). PROSA (25) and PROCHECK (26) were used to check the quality of the predicted structures for further analysis. The modeled structures were validated from the percentage of residues in the Ramachandran favored region and the Z-score obtained from PROSA. The protein functional domains were determined from the InterPro server (http://www.ebi.ac.uk/interpro/), which analyzes the function of proteins by organizing them into families, domain prediction, and critical sites. InterPro uses predictive models or signatures provided by different databases to classify the proteins (27).

### Thermodynamic stability assessment

The effect of point mutations on the stability of the proteins was evaluated using the DUET online tool. The tool combines two complimentary approaches including site-directed mutator (SDM) and cutoff scanning matrix (mCSM). The results obtained by these approaches were combined by support vector machines. The tool was trained on experimental thermodynamic data sets derived from the PROTHERM database. DUET showed the stability of proteins through changes in the unfolding Gibbs free energy (ΔΔG kcal/mol) between the WT and the mutant proteins. Additionally, the mutations are classified as destabilizing (ΔΔG < 0) or stabilizing (ΔΔG > 0) (28).

## RESULTS

### AST status of the clinical isolates and the determination of MIC for colistin by BMD

When we analyzed the AST profile, we found that 1,212 isolates (blood, *n* = 313 and ETA, *n* = 899) were carbapenem resistant. Of the remaining two isolates, one from ETA was pan-susceptible (AB01, aAccession no. CP040080), while the other isolate (AB025, accession no. CP050432) was found to be resistant to cephalosporins, fluoroquinolones, aminoglycosides, tetracyclines, tigecycline, and trimethoprim–sulfamethoxazole and was identified as multi-drug resistant. Thirty-four isolates from blood (10.8%) and 74 isolates from ETA (8.2%) were colistin resistant. The colistin MIC range, MIC_50_, and MIC_90_ of the blood and ETA isolates are tabulated (Table 1).

**TABLE 1:** MIC range, MIC_50_, and MIC_90_ of colistin for clinical isolates of *A. baumannii*

Colistin MIC (µg/ml)	Blood (*N* = 314)	Respiratory (*N* = 900)
MIC range	0.12–64	0.12–64
MIC_50_	0.5	1
MIC_90_	2	2

### Evaluation of colistin resistance mechanisms by WGS

Sequencing analysis of both carbapenem/colistin-resistant *A. baumannii* (CR-ColRAB) (*n* = 115) and carbapenem-resistant colistin-susceptible *A. baumannii* (CR-ColSAB) (*n* = 92) revealed the presence of multiple amino acid substitutions in the *lpx* genes. We found single amino acid substitution in the *lpxA* gene (Y131H), four in the *lpxC* gene (C120R, P148S, F230Y, and N287D) and seven in the *lpxD* gene (Q4K, V63I, V93I, E117K, G166S, T287I, and S299P) when compared with the ATCC 17978 reference strain. Three amino acid substitutions including Q4K, V63I, and E117K were identified in *lpxD*, in both CR-ColSAB and CR-ColRAB isolates. Y131H, C120R, and N287D substitutions were identified in all the sequenced isolates and the three reference strains. It is important to mention that amino acid substitutions such as V93I, G166S, T287I, and S299P in the *lpxD* were not previously found. Similarly, we identified two substitutions in the *lpxC* gene (P148S and F230Y) that were never reported before. Sequencing of *pmrAB* genes identified a single amino acid substitution in *pmrA* (M12I) and seven amino acid substitutions in *pmrB* (A138T, L168S, A226T, V300E, G315A, P360Q, and A444V). Of note, only two mutations, T7I and V383I, were identified in the *pmrC* gene specific to colistin-resistant *A. baumannii*.

The 28 complete genomes of *A. baumannii* were characterized for other colistin resistance mechanisms, such as the insertional inactivation of *lpxA* or *lpxC* by IS*Aba11* and the presence of IS*Aba1* upstream of *eptA*. Two CR-ColSAB blood isolates, AB016 (CP040259) and AB017 (CP050385), showed the presence of IS*Aba11*, but no disruption of *lpxA* or *lpxC* was observed. At least one copy of the *eptA* gene was identified among the 10 and 6 genomes of CR-ColSAB from blood and ETA, respectively. In contrast, more than one copy of *eptA* gene was present in 12 genomes (seven CR-ColSAB and four CR-ColRAB isolates from blood and one CR-ColRAB isolate from ETA).

**Fig 1:**
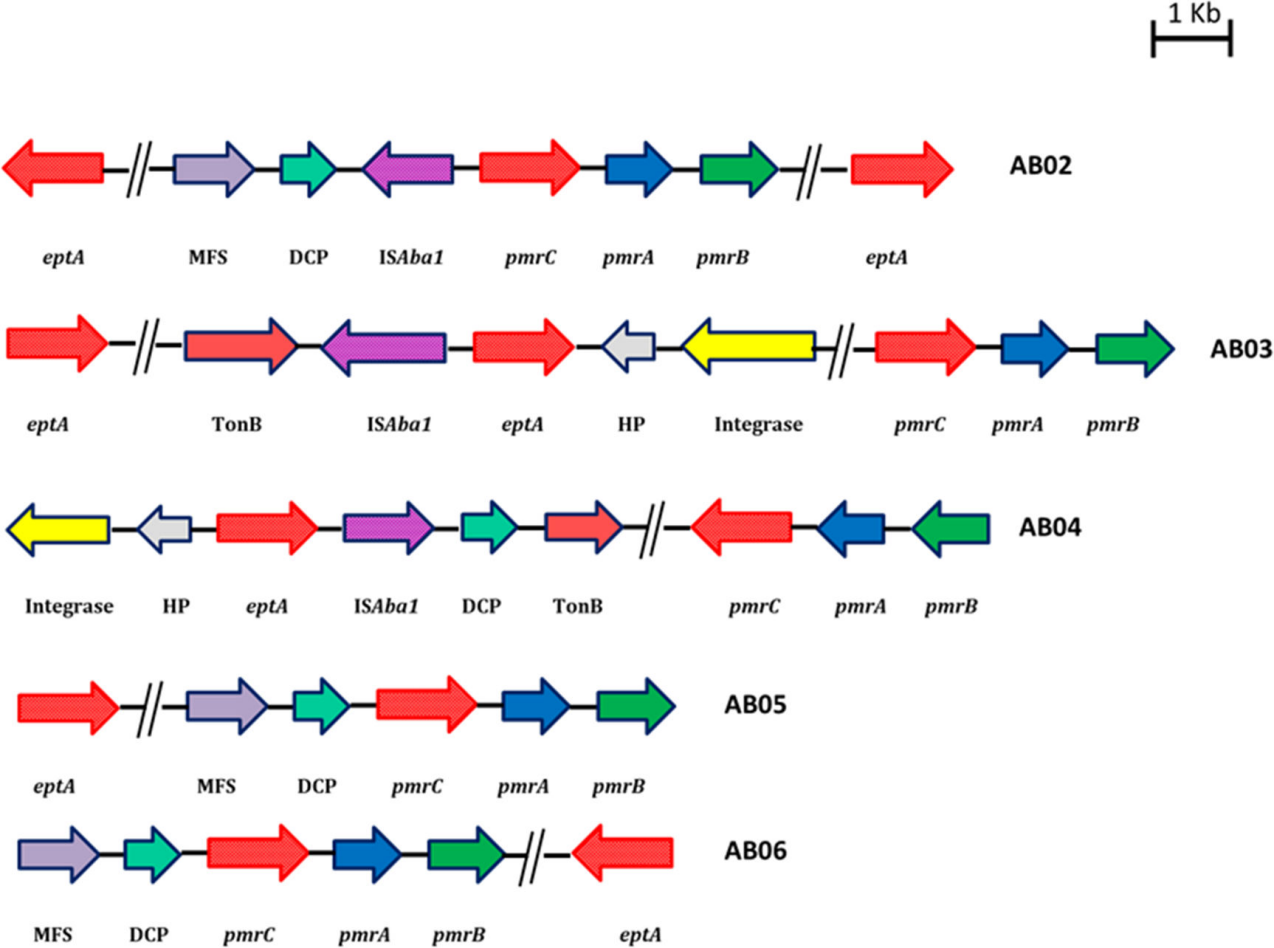
Genetic arrangement of *pmrC/eptA* with upstream presence of IS*Aba1* among complete genomes of colistin-resistant *A. baumannii* (AB02, AB03, and AB04). The direction of the arrow represents the orientation; *pmrC*/*eptA* is shown as red arrows, IS*Aba1* as purple arrows, *pmrA* as blue arrows, and *pmrB* as green arrows. The genetic arrangement of isolates AB05 and AB06 has *eptA* and *pmrC* without IS*Aba1*.

According to Fig. 1, IS*Aba1* is upstream of *pmrC* in AB02, upstream of *eptA* in AB03, but downstream of *eptA* in AB04. In addition, IS*Aba1* is in the reverse orientation compared to *pmrC*/*eptA* in AB02 and AB03 but is in the same orientation as *eptA* in AB04 (but downstream) (Fig. 1).

**Fig 2:**
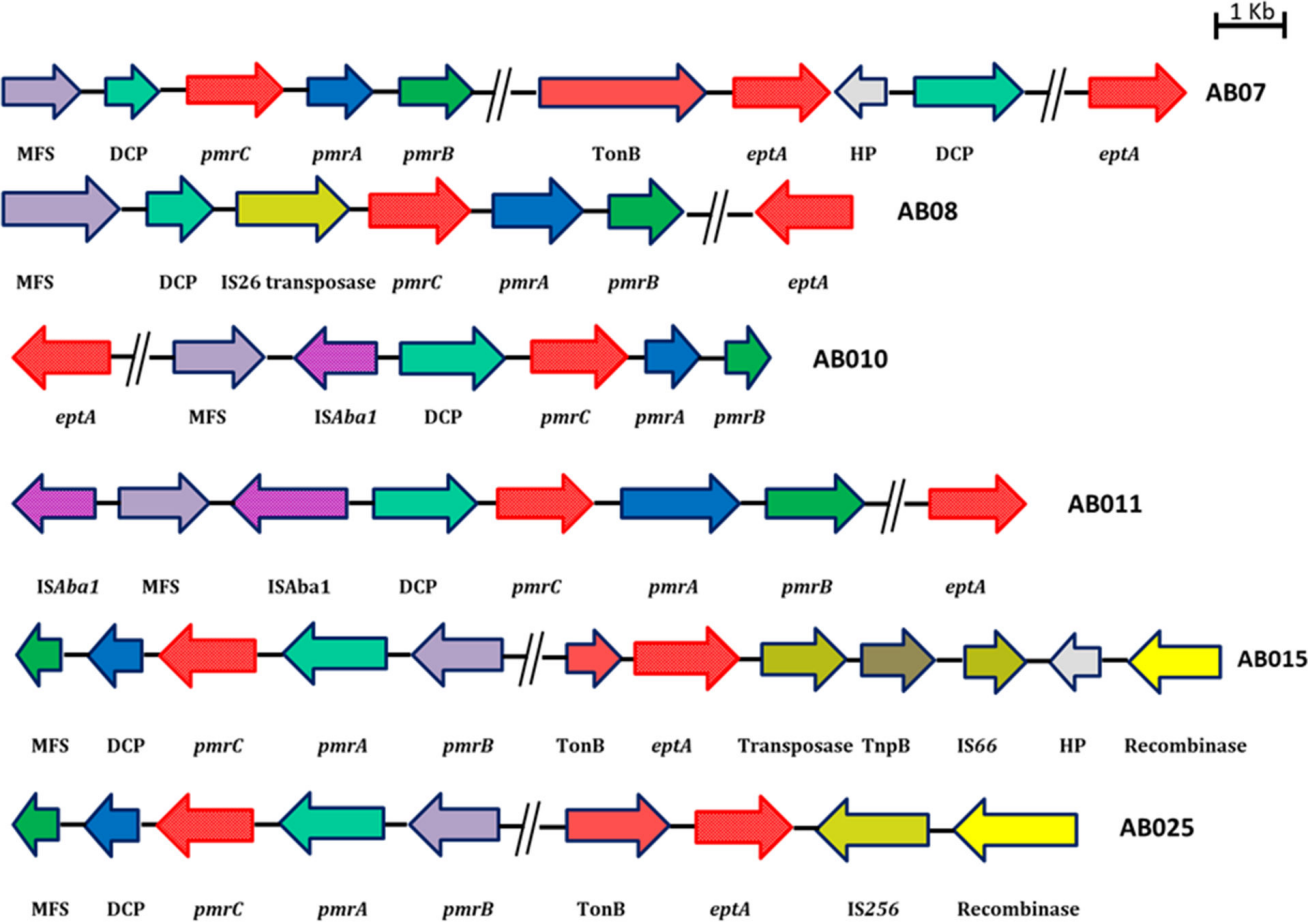
Genetic arrangement of *pmrC*/*eptA* among complete genomes of colistin-susceptible *A. baumannii*. The genetic arrangement of AB07-*pmrC*/*eptA* without insertion element; AB08-*pmrC* with IS26 transposase; AB010 and AB011 with IS4 transposase but not upstream or downstream of *pmrC/eptA*; AB015-*eptA* with IS66 transposase and AB025-*eptA* with IS256 transposase. The direction of arrow represents the orientation. *pmrC/eptA* is shown by red arrows; IS26, IS6, IS256, and IS66 transposases as light green arrows; *pmrA* as blue arrows; and *pmrB* as green arrows.

Two of the CR-ColSAB isolates from blood had IS*Aba1* insertion but not to the upstream of the *pmrC* or *eptA* genes (AB010 [CP040053] and AB011 [CP040056]), whereas three CR-ColSAB isolates from blood (AB08 [CP038500], AB015 [CP050403], and AB025 [CP050432]) have other family transposases (Fig. 2).

Interestingly for one of the CR-ColRAB isolates, AB06, we detected a single amino acid substitution in the *lpxA* gene (Y131H), two substitutions in the *lpxC* gene (C120R and N287D), and two substitutions in the *lpxD* gene (P148S and G315D); however, we did not detect other known mutations or mechanisms that result in colistin resistance. The summary of the findings of CR-ColSAB and CR-ColRAB isolates are listed in Table 2.

**TABLE 2:** Characterization of various colistin resistance mechanisms among complete genomes of *A. baumannii* (*n* = 28)

Isolate ID (accession number)	Colistin susceptibility	Mutation profile*^a^*						IS*Aba1-eptA/pmrC*	Insertional inactivation of *lpxA/lpxC* by IS*Aba11*	Complete *lpxACD* genes	ST (Oxford/ Pasteur)
		lpxA	lpxC	lpxD	pmrA	pmrB	pmrC				
AB01 (CP040080)	Pan-susceptible	Y131H	C120R, N287D	Absent	Absent	Absent	H39Y, I58V, I131V, T142S, F166L, I228V, R348K, A370S	Absent	Absent	Present	2,439/285
AB02 (CP035672)	Resistant	Y131H	C120R, N287D	E117K	Absent	A444V	F166L, I228V, R348K, A370S, K531T	Present	Absent	Present	848/2
AB03 (CP050388)	Resistant	Y131H	C120R, N287D, D159N	Q4K	Absent	Absent	I228V, R348K	Present	Absent	Present	848/2
AB04 (CP040040)	Resistant	Y131H	Absent	E117K	M12I	A138T, A444V	I228V, R348K	Present	Absent	*lpxC* absent	848/2
AB05 (CP040047)	Resistant	Y131H	C120R, N287D	E117K	M12I	A138T, A444V	I228V, R348K	Absent	Absent	Present	848/2
AB06 (CP040050)	Resistant	Y131H	C120R, N287D, P148S	Absent	Absent	G315D	T7I, I58V, K213R, I228V, R348K, V383I	Absent	Absent	Present	2,440/622
AB07 (CP035930)	Susceptible	Y131H	C120R, N287D, P148S	E117K	Absent	A444V	F166L, I228V, R348K, A370S, K531T	Absent	Absent	Present	349/2
AB08 (CP038500)	Susceptible	Y131H	C120R, N287D	Q4K	Absent	Absent	I58V, I131N, I228V, D298G, R348K	Absent	Absent	Present	585/10
AB09 (CP038644)	Susceptible	Y131H	C120R, N287D	Absent	Absent	Absent	I58V, I131V, I228V, I342T, R348K	Absent	Absent	Present	1,089/85
AB10 (CP040053)	Susceptible	Y131H	C120R, N287D	Q4K	Absent	Absent	I58V, I131N, I228V, D298G, R348K	Absent	Absent	Present	2,392/586
AB11 (CP040056)	Susceptible	Y131H	C120R, N287D, D159N	Q4K	Absent	Absent	I58V, I131N, I228V, D298G, R348K	Absent	Absent	Present	2441/575
AB12 (CP040084)	Susceptible	Y131H	C120R, N287D	E117K	Absent	A444V	F166L, I228V, R348K, A370S, K531T	Absent	Absent	Present	1,052/2
AB13 (CP040087)	Susceptible	Y131H	C120R, N287D, P148S	E117K	Absent	A444V	I228V, R348K	Absent	Absent	Present	349/2
AB14 (CP040259)	Susceptible	Y131H	C120R, N287D	Absent	Absent	Absent	I58V, I131V, I228V, Q232H, R348K, K514N	Absent	Absent	Present	1,388/25
AB15 (CP050385)	Susceptible	Y131H	C120R, N287D	E117K	Absent	A444V	I58V,I131V, I228V, Q232H, R348K, K514N	Absent	Absent	Present	691/25
AB16 (CP050523)	Susceptible	Y131H	C120R, N287D	V63I, G166S	Absent	Absent	F166L, I228V, R348K, A370S, K531T	Absent	IS*Aba11* present without disruption	Present	218/2
AB17 (CP050400)	Susceptible	Y131H	C120R, N287D	Absent	Absent	Absent	F166L, I228V, R348K, A370S, K531T	Absent	IS*Aba11* present without disruption	Present	369/2
AB18 (CP050390)	Susceptible	Y131H	C120R, N287D	E117K	Absent	A444V	F166L, I228V, R348K, A370S, K531T	Absent	Absent	Present	208/2
AB19 (CP050410)	Susceptible	Y131H	C120R, N287D, P148S	E117K	Absent	A444V	F166L, I228V, R348K, A370S, K531T	Absent	Absent	Present	451/2
AB21 (CP050415)	Susceptible	Y131H	Absent	E117K	M12I	A138T, A444V	F166L, I228V, A370S, K531T	Absent	Absent	Present	195/2
AB21 (CP050415)	Susceptible	Y131H	C120R, N287D	E117K	M12I	A138T, A444V	I58V, I131N, I228V, D298G, R348K	Absent	Absent	Present	391/10
AB22 (CP050425)	Susceptible	Y131H	C120R, N287D, P148S	Absent	Absent	G315D	I58V, I131N, I228V, D298G, R348K	Absent	Absent	Present	391/10
AB24 (CP051474)	Susceptible	Y131H	C120R, N287D	Q4K	Absent	Absent	I58V,V118F, I131V, V151A, I228V, D298G, R348K	Absent	Absent	Present	447/10
AB24 (CP051474)	Susceptible	Y131H	C120R, N287D	Absent	Absent	Absent	F166L, I228V, R348K, A370S, K531T	Absent	Absent	Present	195/2
AB25 (CP050526)	Susceptible	Y131H	C120R, N287D	Q4K	Absent	Absent	F166L, I228V, R348K, A370S, K531T	Absent	Absent	Present	451/2
AB27 (CP050421)	Susceptible	Y131H	C120R, N287D	E117K	Absent	A444V	F166L, I228V, R348K, A370S, K531T	Absent	Absent	Present	195/2
AB27 (CP050421)	Susceptible	Y131H	C120R, N287D, P148S	E117K	Absent	A444V	F166L, I228V, R348K, A370S, K531T	Absent	Absent	Present	451/2
AB28 (CP050403)	Susceptible	Y131H	C120R, N287D	V63I, G166S	Absent	Absent	I58V, I131V, I228V, Q232H, R348K, K514N	Absent	Absent	Present	231/1

^a^ Underlined text indicates novel SNPs identified in this study.

Finally, it is important to mention that among the colistin-resistant isolates that we tested, we did not identify any of the reported plasmid-mediated colistin resistance determinant, such as *mcr*-1 or its homologs.

### Determination of MLST

Our analyses with MLST Finder revealed that both CR-ColSAB and CR-ColRAB isolates were categorized into four ICs (IC2, IC7, IC8, and IC1), one clonal complex (CC) (CC862), and singletons. Among the CR-ColRAB, IC2 was the major lineage (*n* = 79) followed by IC7 (*n* = 6), IC8 (*n* = 3), CC862 (*n* = 2), and singletons (*n* = 2). Similar to CR-ColRAB, majority of the CR-ColSAB isolates belonged to IC2 (*n* = 90) followed by singletons (*n* = 7), IC8 (*n* = 6), IC7 (*n* = 5), CC862 (*n* = 4), and IC1 (*n* = 3).

We found lineage-specific amino acid substitutions such as Q4K, V63I, and E117K within *lpxD* gene, among isolates belonging to IC8, IC7, and IC2 clades, respectively. We also identified that isolates belonging to the IC2 clade carry substitutions, such as M12I in the *pmrA* gene and the combination of A138T plus A444V within the *pmrB* gene. The effect of mutations was evaluated through *in silico* analysis.

### Structural prediction of the protein and the impact of SNPs on stability

**Fig 3:**
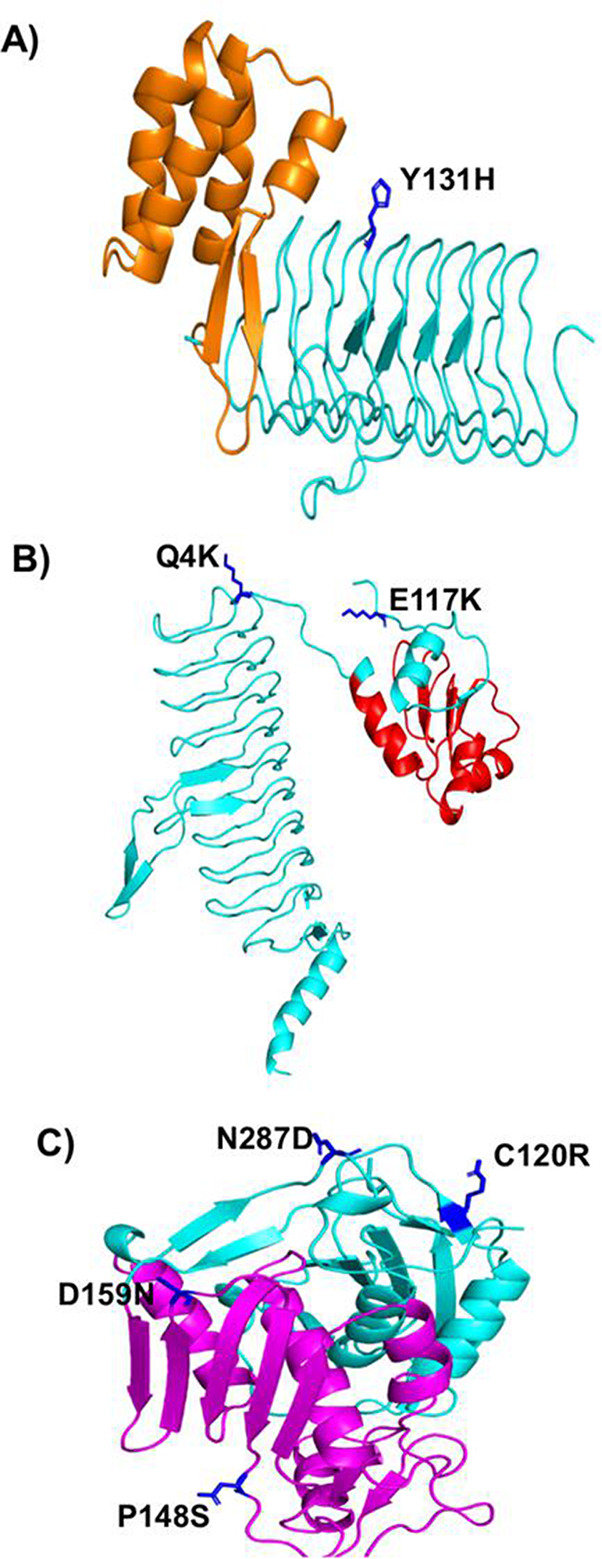
Mapping of mutations in LpxACD. (A) LpxA, domain UDP *N*-acetylglucosamine *O*-acyltransferase in orange; (B) LpxC, domain ribosomal protein S5 domain 2-type fold in red; and (C) LpxD, domain UDP-3-*O*-(3-hydroxymyristoyl) glucosamine *N*-acyltransferase, non-repeat region in magenta. Mutated amino acids are represented in stick model, blue color.

**Fig 4:**
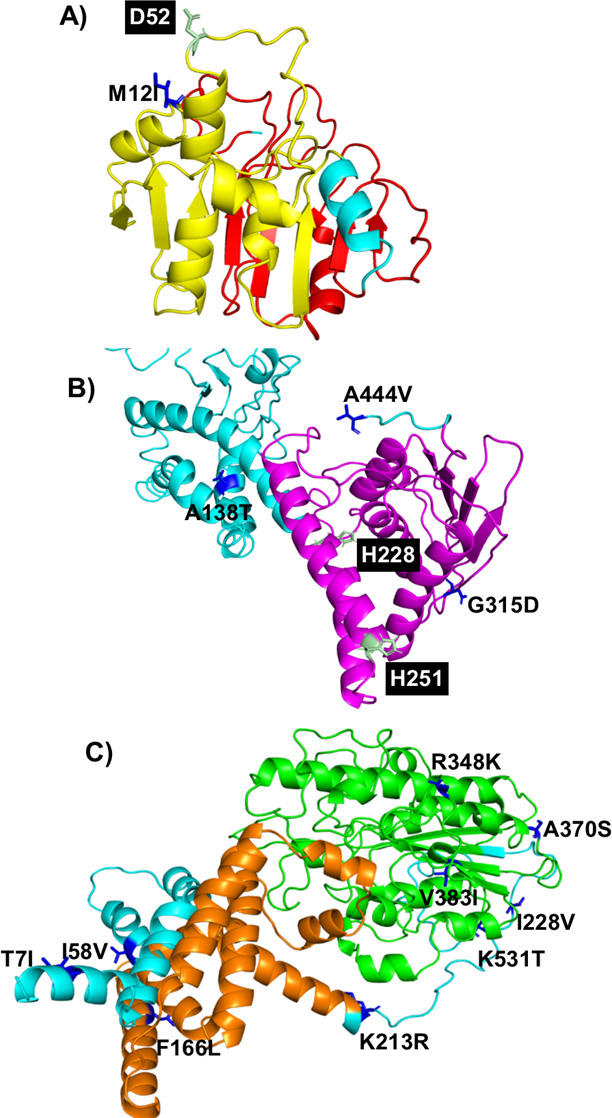
Mapping of mutations in PmrCAB. (A) PmrA, domain signal transduction response regulator in red and domain OmpR/PhoB-type DNA binding in yellow; (B) PmrB, histidine kinase domain in magenta; and (C) PmrC, domain phosphoethanolamine transferase in orange and domain sulfatase in green. Key regulatory amino acids are represented in stick model, pale green color, and mutated amino acids are represented in stick model, blue color.

The 3D structural information for PmrB and PmrC was unavailable in the PDB database. Thus, we modeled the structures of PmrB and PrmC from their primary sequences using I-TASSER. The primary analysis generated five different structural models. The model with a C-score of −2.32 in the PmrB protein and the model with a C-score of 1.13 in the PmrC were selected as these models displayed higher confidence based on the C-scores. We further validated these structures individually. The modeled structures of PmrB and PmrC proteins showed >95% of residues in the favored and allowed regions, and their corresponding Z-scores were −5.56 and −5.96, respectively. Therefore, these structures were selected for further mapping analysis. The SNPs identified in this study were mapped onto the respective protein structures as shown in Fig. 3 (LpxACD) and Fig. 4 (PmrCAB).

The domains of LpxACD and PmrCAB, predicted by the InterPro server, are listed in Table 3. The possible structural impact of the mutations observed in this study was examined and is given in Table 4. Based on domain screening, in LpxA, even though the mutation was not present in the UDP *N*-acetylglucosamine *O*-acyltransferase domain, Y131H was found to be destabilizing. In LpxC, two SNPs (P148S and D159N) were mapped in the ribosomal protein domain that might destabilize LpxC, whereas the other SNPs (C120R in the N-terminal and N287D in the C-terminal) were found to be stabilizing the LpxC structure. Both SNPs (E117K and Q4K) in LpxD and an SNP (M12I) in PmrA were not mapped in their domain region and might stabilize the protein structures. Interestingly, we have noted that all SNPs (A138T, G315D, and A444V) in PmrB destabilizes the protein although A138T substitution was not present in the histidine kinase domain. In PmrC, nine SNPs were predicted. Among these, one SNP (F166L) was mapped in the phosphoethanolamine domain, while the other SNPs (R348K, A370S, and V383I) were mapped in the sulfatase. We found that only two SNPs (T7I and R348K) might be stabilizing the structure, whereas all other SNPs have a destabilizing effect. The exact relevance of these SNPs on the protein activity needs to be biochemically evaluated.

**TABLE 3:** Domains of LpxACD and PmrCAB predicted by the InterPro server

Protein	InterPro ID	Domain	Position
LpxA	IPR029098	UDP *N*-acetylglucosamine *O*-acyltransferase	179–261
LpxC	IPR020568	Ribosomal protein S5 domain 2-type fold	133–277
LpxD	IPR020573	UDP-3-*O*-(3-hydroxymyristoyl) glucosamine *N*-acyltransferase, non-repeat region	27–92
PmrA	IPR001789	Signal transduction response regulator, receiver domain	2–116
	IPR001867	OmpR/PhoB-type DNA-binding domain	129–223
PmrB	IPR005467	Histidine kinase domain	225–437
PmrC	IPR012549	Phosphoethanolamine transferase	61–210
	IPR000917	Sulfatase	239–530

**TABLE 4:** Predicted effect of protein stability in the presence of amino acid mutations in the LpxACD and PmrCAB proteins

Protein	Mutation	ΔΔG mCSM (kcal/mol)	ΔΔG SDM (kcal/mol)	ΔΔG DUET (kcal/mol)	Stability
LpxA	Y131H	−0.803 (Destabilizing)	0.02 (Stabilizing)	−0.497 (Destabilizing)	Destabilizing
LpxC	C120R	−0.417 (Destabilizing)	0.02 (Stabilizing)	0.049 (Stabilizing)	Stabilizing
	P148S	−1.28 (Destabilizing)	−0.84 (Destabilizing)	−1.125 (Destabilizing)	Destabilizing
	D159N	−0.688 (Destabilizing)	0.17 (Stabilizing)	−0.425 (Destabilizing)	Destabilizing
	N287D	0.128 (Stabilizing)	0.07 (Stabilizing)	0.497 (Stabilizing)	Stabilizing
LpxD	E117K	0.492 (Stabilizing)	−0.27 (Destabilizing)	0.665 (Stabilizing)	Stabilizing
	Q4K	0.009 (Stabilizing)	0.38 (Stabilizing)	0.448 (Stabilizing)	Stabilizing
PmrA	M12I	−0.275 (Destabilizing)	0.08 (Stabilizing)	0.284 (Stabilizing)	Stabilizing
PmrB	A138T	−1.202 (Destabilizing)	−2.62 (Destabilizing)	−1.359 (Destabilizing)	Destabilizing
	G315D	−0.953 (Destabilizing)	−0.88 (Destabilizing)	−0.776 (Destabilizing)	Destabilizing
	A444V	−0.331 (Destabilizing)	0.0 (Destabilizing)	−0.009 (Destabilizing)	Destabilizing
PmrC	T7I	−0.363 (Destabilizing)	1.04 (Stabilizing)	0.228 (Stabilizing)	Stabilizing
	I58V	−0.555 (Destabilizing)	−0.18 (Destabilizing)	−0.207 (Destabilizing)	Destabilizing
	F166L	−0.806 (Destabilizing)	0.57 (Stabilizing)	−0.51 (Destabilizing)	Destabilizing
	K213R	−0.921(Destabilizing)	0.72 (Stabilizing)	−0.456 (Destabilizing)	Destabilizing
	I228V	−1.22 (Destabilizing)	0.0 (Destabilizing)	−0.956 (Destabilizing)	Destabilizing
	R348K	−0.19 (Destabilizing)	0.06 (Stabilizing)	0.055 (Stabilizing)	Stabilizing
	A370S	−1.122 (Destabilizing)	−0.9 (Destabilizing)	−1.117 (Destabilizing)	Destabilizing
	V383I	−0.64 (Destabilizing)	−0.6 (Destabilizing)	−0.39 (Destabilizing)	Destabilizing
	K531T	−0.483 (Destabilizing)	−0.32 (Destabilizing)	−0.3 (Destabilizing)	Destabilizing

## DISCUSSION

In this study, we found 8%–11% prevalence of colistin-resistant *A. baumannii*. Similarly, reports from other countries also suggest increased colistin resistance rates. For instance, 16.7% from Bulgaria, 19.1% from Spain, and 27% from Greece have been reported (29–32). Therefore, these increasing prevalence of colistin resistance rate highlights the importance of routine testing and understanding of colistin resistance mechanisms (3).

Previous studies reported genetic polymorphisms within the *lpxACD* and *pmrCAB* operons that led to altered or loss of LPS/lipooligosaccharide production (9, 10). In this study, we identified several SNPs within the *lpxACD* operon. The amino acid substitutions identified in this study in the *lpxD* gene (Q4K, V63I, and E117K) are similar to the previous studies (33, 34). Recent studies suggest that mutations in the *pmrB* gene, which encodes for histidine kinase, are one of the major contributors for colistin resistance in *A. baumannii* (31, 33, 35, 36).

Moffatt and colleagues have reported that inactivation of the *lpxA* or *lpxC* genes caused by insertion of IS*Aba11* elements resulted in the loss of LPS production that confers resistance to colistin (10). In addition, inactivation of the *lpxC* gene due to insertion of IS*Aba11* has also been reported (37). Though the current study showed the presence of IS*Aba11* in two CR-ColSAb isolates, disruption of *lpxA* or *lpxC* was not observed.

A single substitution M12I was detected in the *pmrA* gene, which encodes for the response regulator of the pmrCAB TCS. This substitution is thought to be associated with colistin heteroresistance (38). However, we have not detected other known substitutions such as G54E within the *pmrA* gene, which is reported to be associated with conferring high colistin resistance in *A. baumannii* either alone or in combination with other genes (36, 39). In a recent study, it has been described that several substitutions, including G54E identified within the receiver domain of the *pmrA* response regulator, are responsible for colistin resistance in *A. baumannii*. Furthermore, G54E substitution alone or in combination with mutations in other genes can confer significantly high colistin resistance up to >256 or 512 µg/mL in *A. baumannii* (40). Previous studies detected A138T substitution in *pmrB* in addition to other substitutions (31, 33, 36). Also, a recent study by Srisakul et al. (41) found S14P and A138T in *pmrB* and reported that it could be correlated with colistin resistance. However, the current study identified only A138T, and the exact effect of this particular substitution needs further investigation.

Gerson and colleagues have reported the presence of additional substitutions in both the *lpxD* and *pmrB* genes (36). Interestingly, we found that similar substitutions in the *lpxD* gene (such as E117K) co-occurred with substitution in the *pmrB* gene (A138T and A444V) while analyzing CR-ColRAB and CR-ColSAB isolates. A similar observation was also reported by Zafer et al. (42). These findings suggest that not all substitutions are associated with colistin resistance, and this highlights the importance of considering their genetic background in addition to SNPs. Thus, further investigation on the role of these substitutions in lipid A modification and the resulting colistin-susceptible phenotype is required. It is evident that colistin resistance mechanisms in *A. baumannii* are much more complicated than imagined.

The *pmrC* gene encodes for pEtN transferase, which is necessary for LPS biogenesis (38). Lesho et al. showed the presence of an alternate gene, *eptA,* with pEtN transferase activity among the clinical isolates of *A. baumannii* (43). The length of the *pmrC* and *eptA* genes is approximately 1,650 bp, and it was found that they are homologous proteins with 93% amino acid identity thereby suggesting similar enzymatic activity (7). Two earlier reports have suggested that in the absence of the *pmrA* gene-mediated expression of *pmrC*, transposition of an insertion element, such as IS*Aba1*, might lead to overexpression of the highly similar pEtN transferase, EptA, which allows addition of pEtN to lipid A and results in colistin resistance among the clinical isolates of *A. baumannii* (3, 7). We found the presence of *eptA* in both CR-ColRAB and CR-ColSAB isolates similar to previous reports (3, 7, 8, 43). However, the presence of the IS*Aba1* element to the upstream region of the *pmrC/eptA* loci was identified only among the CR-ColRAB isolates, which might lead to overexpression of pEtN transferase activity and increased colistin resistance. This observation is in agreement with previous studies (3, 8, 43). Recently, it has been reported that the disruption of the *eptA* gene by insertion of the IS*Aba125* element or increased expression of the *eptA* gene due to the insertion of IS*Aba1* in the reverse orientation to the upstream of *eptA* is also associated with colistin resistance (3). Though the difference in the orientation or the position of the IS*Aba1* insertion with respect to the *eptA* locus was identified in this study, further studies are warranted to understand the impact on the expression on *ept*A.

In summary, our study provides characterization of multiple resistance mechanisms that could be responsible for the emergence of colistin resistance among *A. baumannii* clinical isolates. Previously reported amino acid substitutions as well as other substitutions that are not described previously within the *lpxD*, *pmrA,* and *pmrB* genes were identified in this study isolates. In particular, the presence of E117K in the *lpxD* gene along with A138T/A444V in the *pmrB* gene suggests a novel synergistic activity for the occurrence of colistin resistance.

In this study, two colistin-susceptible isolates harbored IS*Aba11,* and studies to understand the role of IS*Aba11* toward colistin resistance are essential. Though the altered expression pattern of *pmrC* and addition of pEtN transferase to the lipid A are regulated by PmrAB TCS, this may not be the sole contributor of colistin resistance. The presence of the additional *eptA* gene encoding pEtN transferase and the insertion of the IS*Aba1* element to the upstream of the *eptA/pmrC* loci among the colistin-resistant isolates might be associated with colistin resistance. The exact resistance mechanism that contributes to colistin resistance was not elucidated for two colistin-resistant isolates (AB05 and AB06) in this study and requires further investigations including transcriptomic analysis.

It must be noted that most of the amino acid substitutions in *lpxACD* and *pmrCAB* identified from our study were present both in colistin-resistant as well in susceptible isolates except the substitutions of T7I and V383I in the *pmrC* gene that are present only in the colistin-resistant isolate, AB06. Even though both substitutions were not present in the functional domain of the protein, V383I destabilizes the PmrC protein structure, whereas T7I stabilizes the protein. To the best of our knowledge, these two substitutions within the *pmrC* gene are novel and not reported elsewhere. Similarly, we observed certain amino acid substitutions in the *lpxD* (V63I and G166S) and the *pmrC* (V118F, I131V, I131N, V151A, Q232H, D298G, I342T, K531T, and K514N) genes that are present only in colistin-susceptible isolates.

With respect to the epidemiologic aspect of the CR-ColSAB and CR-ColRAB observed in this study, we found several lineage-specific mutations, such as Q4K that belongs to IC8, V63I in IC7, and E117K/M12I/A138T in IC2 clades. Earlier studies reported IC2 as the predominant lineage involved in outbreaks (8, 13). We consider these as novel findings because of the associations of the SNPs with the clones, which are not reported earlier. Overall, the present study highlights the diversity of colistin resistance mechanisms among the clinical isolates of *A. baumannii*.

## Data Availability

The data corresponding to WGS project have been deposited to DDBJ/ENA/GenBank under the BioProject numbers PRJNA603876, PRJNA604897, PRJNA610496, and PRJNA610503. The complete genome project has been deposited to DDBJ/ENA/GenBank under the accession numbers CP040080, CP035672, CP050388, CP040040, CP040047, CP040050, CP038500, CP038644, CP040053, CP040056,CP040084, CP040087, CP050421, CP050403, CP040259, CP050385, CP050523, CP050400, CP050390, CP050410, CP050412, CP050415, CP050425, CP050432, CP051474, CP050526, and CP050401.
